# Resistant Potato Starch Supplementation Increases the Serum Levels of Choline and Sphingomyelins Without Affecting Trimethylamine Oxide Levels

**DOI:** 10.3390/metabo15100662

**Published:** 2025-10-11

**Authors:** Jason R. Bush, Jun Han, David R. Goodlett

**Affiliations:** 1 MSP Starch Products Inc., Carberry, MB R0K 0H0, Canada; 2 The University of Victoria—Genome British Columbia Proteomics Centre, and Division of Medical Sciences, University of Victoria, Victoria, BC V8Z 7X8, Canada; hanjun@proteincentre.com (J.H.); goodlett@uvic.ca (D.R.G.)

**Keywords:** prebiotic, resistant potato starch, choline, betaine, acetylcholine, trimethylamine, trimethylamine oxide, sphingomyelin, hydroxysphingomyelin, phosphocholine, glycerophosphocholine

## Abstract

**Background/Objectives:** The prebiotic effect of resistant potato starch (RPS) has been demonstrated, but the role of this nutrient in choline metabolism and the production of microbially modified choline-derived toxins is unknown. **Methods:** We performed post hoc analysis comparing changes in choline and related metabolites in serum from baseline to the week 4 time point in a human clinical trial evaluating daily consumption of 3.5 g RPS versus a placebo. **Results:** Choline levels increased in the RPS consuming group, while levels of trimethylamine decreased and levels of the cardiovascular toxin trimethylamine oxide were unaffected by RPS consumption. Increases in choline were positively correlated with increases in *Akkermansia* in the gut. Oxidation of choline to betaine was unaffected by RPS, as was acetylcholine metabolism. Levels of various saturated even acyl chain and hydroxylated acyl chain sphingomyelins were increased in RPS consuming participants, and levels of phospholipid degradation products phosphocholine and glycerophosphocholine were decreased. **Conclusions:** These data suggest that RPS enhances choline absorption without increasing TMAO and stimulates the incorporation of choline into sphingomyelins containing saturated even acyl chains and hydroxylated acyl chains. Future studies assessing the physiological consequences, such as cognitive or neurological benefits, of enhanced choline absorption and sphingomyelin levels in people consuming RPS are warranted.

## 1. Introduction

**Figure 1:**
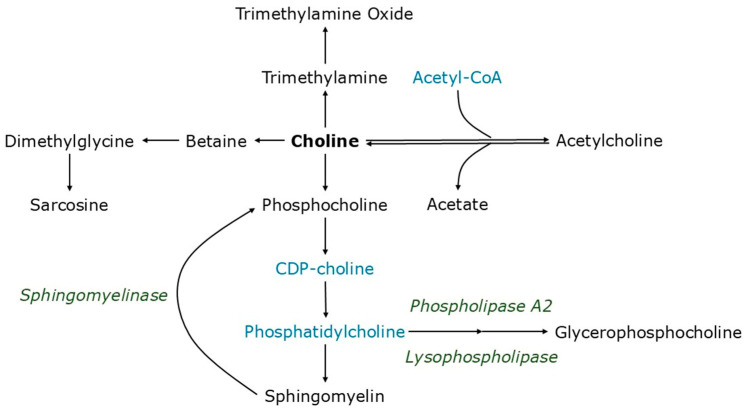
Schematic depicting the fate of choline and choline intermediates. Metabolites in black were captured in the current analysis and are presented here. Metabolites in blue were not captured. Enzymes in green catalyze catabolic reactions of phospholipids.

Choline is an essential nutrient for human health and development, and is involved in the synthesis of phospholipids and acetylcholine, and for DNA methylation (Figure 1) [1,2]. Humans can synthesize choline to some extent, but this production is incapable of meeting the body’s needs [3]. Choline can be obtained from a variety of foods, with whole eggs, liver, meat, and whole grains providing excellent sources [4]. Choline deficiency can lead to muscle and liver damage, in some cases progressing into non-alcoholic fatty liver disease (NAFLD) [3,5]. Furthermore, certain populations are particularly at risk of choline deficiency, including pregnant women and nursing mothers [6,7], those with polymorphisms in the genes encoding proteins important for choline metabolism [8,9,10], people requiring total parenteral nutrition [9,11], those with neurological conditions [9,12,13], and people at risk of developing NAFLD [9,14]. For example, the PRISM study found that 95% of participants consumed less than the adequate intake for choline [6], while meta-analysis of 12 studies found that 11% of pregnant women had adequate choline intake [7]. This has clinical ramifications, with the odds ratio of developing a neural tube defect is 0.17 when comparing mothers whose intake was above the 75% percentile to those whose choline intake was below the 25% percentile [9]. While choline supplementation has not been widely explored in hereditary neurological orders associated with choline metabolism [9], choline intake influences homocysteine levels in people with *MTHFR* mutations, with higher levels (>1100 mg) suppressing plasma homocysteine levels compared to diets containing lower levels (<550 mg/day) [10]. Dietary supplementation with choline is recommended to pregnant women and those trying to conceive, with a combination of dietary intake and supplementation helping this population to meet their daily targets [15].

Choline supplementation and diets enriched with choline-containing foods can also promote adverse health risks. Dietary choline was identified as a risk factor for cardiovascular disease (CVD) and mouse studies demonstrated that microbial transformation of choline enhanced the abundance of the toxic metabolite trimethylamine oxide (TMAO) [16]. Gut bacteria metabolize choline to produce the intermediate metabolite trimethylamine (TMA), which is then converted by the liver into TMAO [17]. Recent studies have emphasized a role for TMAO in CVD, including associations with abdominal aortic aneurysms [18] and atherosclerosis-associated CVD events [19]. It is therefore important that supplementation or dietary interventions meant to improve serum choline levels be balanced against the risk of elevating TMAO, especially in those at risk of developing CVD.

Sphingomyelins (SMs) are a diverse class of phospholipids consisting of a sphingosine base attached to a fatty acid and phosphocholine [20]. Sphingomyelins are synthesized through a complex process: First, serine palmitoyltransferase (SPT) facilitates the condensation of a fatty acid CoA, usually palmitoyl CoA, and L-serine to form 3-ketodihyrosphingosine, a process regulated by SPT subunit composition [21]. Reduction to sphinganine occurs via 3-ketodihydrosphingosine reductase, which leads to ceramide production via various ceramide synthases, depending on acyl chain length [22]. Following the Kennedy pathway, choline is phosphorylated by choline kinase to form phosphocholine, then metabolized to CDP-choline by CTP:phosphocholine cytidylyltransferase, from which phosphatidyl choline forms via condensation with diacylglycerol, in a step that is catalyzed by cholinephosphotransferase [23]. Finally, phosphatidylcholine is transferred to the ceramide via sphingomyelin synthase to form the SM molecule [20]. These phospholipids play an important role in maintaining the integrity of cell membranes, especially in cells like oligodendrocytes that synthesize lipids important for myelination [20]. Hydroxylated forms of SM are particularly abundant in the myelin sheath, as are long chain SMs [23]. Mice lacking the enzyme required for SM hydroxylation develop normally but experience enhanced degeneration of the myelin sheath [24]. Despite their importance, intact SMs cannot be obtained from the diet [25], requiring the body to synthesize sphingomyelin de novo.

Resistant potato starch (RPS) is a Type 2 resistant starch (RS2) that has prebiotic effects [26], including increasing *Bifidobacterium* [27,28,29] and *Akkermansia* [29] in the gut, and improving bowel movement scores [29], decreasing serum histamine levels and markers of intestinal permeability [30] and reducing markers of insulin resistance [31,32] in the host. Supplementing diets with high amylose maize starch (HAMS; another RS2) was shown to increase levels of TMAO in healthy people [33] but had no effect on plasma choline or TMAO levels in chronic kidney disease patients [34]. While RPS and HAMS are both RS2, there are important differences in the amylose-to-amylopectin ratio that likely influence crystalline structure and microbial accessibility and activity [35]. Given the potential risk of TMAO production in people consuming RS as a supplement, we hypothesized that RPS might increase TMAO levels and, therefore, explored the role of RPS in choline metabolism using serum metabolomic data from a clinical trial conducted in healthy adults.

## 2. Materials and Methods

### 2.1. Investigational Product

The resistant potato starch (RPS) used in this study was Solnul^®^ (MSP Starch Products Inc., Carberry, MB, Canada), an unmodified RS2 produced via a proprietary processing method to preserve resistant starch (RS). Solnul^®^ contains a minimum resistant starch content of 60% (AOAC 2002.02), 70% dietary fiber (AOAC 2009.01), and less than 20% moisture (AOAC 930.15), and contains 324 KCal/100 g (calculated based on macronutrient contents). The RPS is composed of granules ranging in size between 15 and 100 μm and has a starch content containing 80% amylopectin (branched glucose polymer with alpha-1,4 and alpha-1,6 linkages) and 20% amylose (linear glucose polymer with mostly alpha-1,4 linkages). The placebo used was a food-grade corn starch derived from waxy maize that contains high levels of naturally occurring amylopectin (Amioca; Ingredion, Brampton, ON, Canada) that is fully digested and has no discernable effects on the gut microbiota [36]. Investigational products were packaged in identical sachets and shipped from MSP Starch Products Inc. (Carberry, MB, Canada) in coded boxes to the contract research organization (Nutrasource, Guelph, ON, Canada).

### 2.2. Study Design

The participants of this study have been described in detail [29]. In brief, healthy adults aged 18–69 years with a body mass index (BMI) of 18.0 to 34.9 kg/m^2^ were recruited and enrolled in the study. Candidate participants with a BMI ≥ 35 kg/m^2^ were excluded. Candidate participants reporting a diagnosis of irritable bowel syndrome, dyspepsia, significant gastrointestinal disorders, or other major diseases were also excluded. This allowed the clinical Principal Investigator to use objective criteria such as diagnosis, medication records, review of recent hospitalizations to determine whether candidates were included or excluded. Those enrolled agreed not to consume any vitamins, minerals, or dietary supplements from 14 days prior to the randomization visit until the study concluded. Participants were counseled to follow their habitual diet throughout the study period and no changes in dietary intake were observed [37].

### 2.3. Clinical Trial Conduct

The study occurred between 30 October 2019 and 6 January 2020 in Guelph, ON, Canada, with participants recruited from the general population in Guelph and the surrounding area. Canadian Shield Ethics Review Board (tracking number 19-10-001; Burlington, ON, Canada) approved the study protocol and the trial was registered at ClinicalTrials.gov (NCT05242913). Written informed consent was obtained from all study participants or their legally authorized representative prior to enrollment into the study following the Declaration of Helsinki and Council for International Organizations of Medical Sciences International Ethical Guidelines and ICH Good Clinical Practice guidelines [29].

The study was a randomized, double-blind, placebo-controlled, parallel-armed clinical trial designed to evaluate the prebiotic effects of daily 3.5 g of RPS (containing 3.5 g of RPS and 3.5 g placebo, with 7 g total carbohydrates), 7 g of RPS, and 7 g of placebo for 4 weeks using fecal samples to estimate gut microbiome composition and bowel movement characterized using the Bristol Stool Form Chart [29]. Participants received sachets without marks identifying the nature of the investigational product and clinic staff were blinded to the investigational product identity. Forty-eight participants completed the study protocol for the 3.5 g RPS (n = 24) and placebo (n = 24) arms. Non-fasting serum samples were collected at baseline and 4 weeks of supplementation and analyzed by targeted metabolomics to quantify circulating levels of polar amines and lipids. Due to commercial interest in the effects of low RPS doses, only the impacts of 3.5 g RPS and placebo on choline metabolism are reported.

### 2.4. Metabolomic Analysis

Polar metabolite analysis, including acetate, has been described in detail [30,32]. Authentic compounds of sphingomyelins (SMs) were acquired from Cayman Chem Inc. (Ann Arbor, ML, USA) and Avanti Polar Lipids, LLC. (Alabaster, AL, USA) and were used for optimization of the precursor-to-product ion transitions of multiple-reaction monitoring mass spectrometry (MRM/MS) by direct infusion of a standard solution of each of the lipids into a Sciex QTRAP 4000 mass spectrometer via a solvent delivery syringe pump. These lipids were also used to help construction of the putative MRM/MS ion transitions of their homologues in each class, for which the authentic compounds were commercially unavailable or not acquired, according to the structures of lipids deposited in the LIPID MAPS database (https://www.lipidmaps.org, accessed on 17 August 2025) or in the Human Metabolome Database (https://hmdb.ca, accessed on 17 August 2025).

A total of 20 µL of human serum from each sample was aliquoted into a 1.5 mL Eppendorf tube and mixed with 400 µL of a mixed solvent of methanol-chloroform (3:1, *v*/*v*). The samples were vortexed for 1 min, ultra-sonicated in an ice water bath for 3 min, and then centrifuged at 21,000× *g* and 5 °C for 10 min inside an Eppendorf 5425R centrifuge (Eppendorf Canada, Mississauga, ON, Canada). The clear supernatants were transferred to 1 mL micro-vials and dried under a nitrogen gas flow. The dried residues were added with 200 µL of methanol-chloroform (1:1, *v*/*v*). After 5 s vortex mixing, 30 s ultra-sonication and 2 min centrifugal clarification at 21,000× *g* and 5 °C, 4 µL aliquots of the clear solutions were injected into a Waters XBridge C8 (2.1 × 50 mm, 2.5 µm; Waters Corp., Milford, MA, USA) column to run liquid chromatography coupled to tandem mass spectrometry (LC-MS/MS) in the dynamic MRM scanning mode on an Agilent 1290 UHPLC instrument hyphenated via an atmospheric pressure electrospray ion source to an Agilent 6495B triple-quadrupole mass spectrometer (Agilent Technologies, Santa Clara, CA, USA). The mass spectrometer was operated in the positive-ion mode for detection of sphingolipids and glycerides using the following parameters: capillary voltage 3500 V, nozzle voltage 1500 V, gas temperature 250 °C, gas flow 15 L/min, nebulizer gas 30 psi, sheath gas temperature 250 °C and sheath gas flow 12 L/min. For chromatographic separations, a binary-solvent mobile phase composed of 2 mM ammonium acetate solution (pH adjusted to 4 with acetic acid) (solvent A) and 2 mM ammonium acetate in a mixture of water-acetonitrile-isopropanol (20:490:490, *v/v/v*, pH adjusted to 4 with acetic acid) (solvent B) was used for gradient elution at 0.4 mL/min and 55 °C. The elution gradient was 0–6 min, 10% to 60% B; 6–22 min, 60% to 100% B and 22–24 min, 100% B. The chromatographic column was re-equilibrated at 10% B for 3 min between injections. For quality control (QC), aliquots of 20 µL serum were pooled from 30 randomly chosen samples. 20 µL aliquots of the pooled serum sample were prepared for the same liquid extraction, along with processing of the batch samples. The resultant QC sample solutions were injected periodically at the beginning, in the middle (every 25 sample solution injections), and at the end of the LC-MRM/MS batch runs to monitor the analytical precision. The LC-MRM/MS data were recorded and subsequently processed using the Agilent MassHunter 10.0 software suite (Agilent Technologies). Peak areas of individual lipids detected in serum were integrated and used for relative quantitation and subsequent statistics. Only the metabolites and lipids detected with their QC coefficients of variation (CVs) of ≤20% were considered for subsequent statistics.

### 2.5. Statistical Analysis

Baseline levels of metabolites in both treatment groups were tested for normality using the Kolmogorov–Smirnov test and four were found to be not normal. Baseline and week 4 metabolite levels were compared within groups using the non-parametric Wilcoxon signed-rank test. Pearson correlation analysis compared choline changes at week 1 and week 4 to changes in *Bifidobacterium* and *Akkermansia* at week 1 and 4 to increase statistical power [36]. Differences were considered significantly different at *p* < 0.05. All comparisons were made using Excel (Version 2409; Microsoft, Redmond, WA, USA).

## 3. Results

### 3.1. Serum Choline Levels

**Figure 2:**
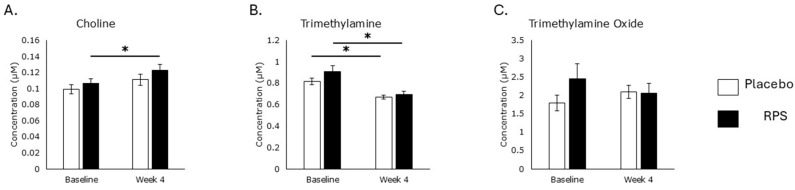
Choline and microbiome-relevant degradation products. RPS consumption led to increases in serum choline levels (*p* = 0.009; (**A**)) and decreases in trimethylamine (*p* = 0.00006; (**B**)). Placebo treatment decreased trimethylamine levels (*p* = 0.00006; (**B**)). Neither RPS (*p* = 0.5) nor placebo (*p* = 0.3) treatment influenced trimethylamine oxide levels (**C**). Wilcoxon signed-rank test; Mean +/− SEM; *, *p* < 0.05.

Choline levels were elevated in the RPS treatment group (*p* = 0.009) but not those consuming the placebo after 4 weeks (*p* = 0.1; Figure 2A). Changes at week 1 and week 4 time points in serum choline were correlated with changes at week 1 and week 4 time points in *Akkermansia* (r = 0.324; *p* = 0.02), a genus associated with intestinal barrier function [38], in participants consuming RPS but not placebo (r = 0.122; *p* = 0.4). Choline may be transformed into trimethylamine (TMA) by the gut microbiota and then into the cardiovascular disease-associated metabolite trimethylamine oxide (TMAO) by the liver [39]. Supplementation with high amylose maize starch, a form of resistant starch type 2, increased TMAO production [33]. We therefore tested whether increased serum choline levels were accompanied by changes in TMA or TMAO. Serum levels of TMA decreased in both the RPS (*p* = 0.00006) and placebo (*p* = 0.00006) treatment groups (Figure 2B). Levels of TMAO tended to rise in the placebo group and fall in the RPS group (Figure 2C).

### 3.2. Choline Oxidation

**Figure 3:**
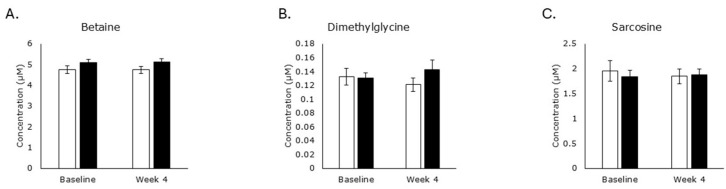
Metabolites associated with the oxidation of choline. Levels of betaine ((**A**); RPS *p* = 1.0; placebo *p* = 1.0), dimethylglycine ((**B**); RPS *p* = 0.7; placebo *p* = 0.5), or sarcosine ((**C**); RPS *p* = 0.6; placebo *p* = 0.6) in serum. White boxes, placebo; Black boxes, RPS; Wilcoxon signed-rank test; Mean +/− SEM.

Choline can be catabolized or incorporated into new molecules via anabolic reactions (Figure 1). To determine the physiological significance of choline increases in the RPS consuming group, we systematically explored each pathway. Levels of betaine (Figure 3A), dimethylglycine (Figure 3B), and sarcosine (Figure 3C) were not affected by either treatment, suggesting that increased choline is not being oxidized in either treatment group.

### 3.3. Acetylcholine Metabolism

**Figure 4:**
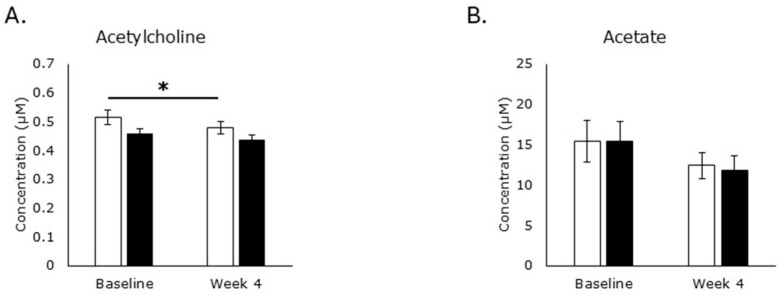
Acetylcholine metabolism. Consumption of the placebo led to a reduction in acetylcholine (*p* = 0.004) but RPS had no effect (*p* = 0.2; (**A**)). Neither RPS (*p* = 0.05) nor placebo (*p* = 0.3) treatment affected acetate levels (**B**) in serum. White boxes, placebo; Black boxes, RPS; Wilcoxon signed-rank test; Mean +/− SEM; *, *p* < 0.05.

Neurons synthesize acetylcholine from choline and acetyl-CoA via choline acetyltransferase, a reaction that is reversed in the synapse via acetylcholinesterase to reform choline and acetate, a process that can also occur outside of the neuromuscular junction [40] (Figure 2). Placebo treatment reduced acetylcholine production (*p* = 0.004), but RPS treatment had no effect (*p* = 0.2; Figure 4A), suggesting that increased choline did not enhance acetylcholine levels in RPS consuming individuals. Furthermore, neither treatment influenced acetate levels (Figure 4B), suggesting that increased choline is not due to elevated acetylcholinesterase activity in the RPS group.

### 3.4. Sphingomyelin Synthesis

**Figure 5:**
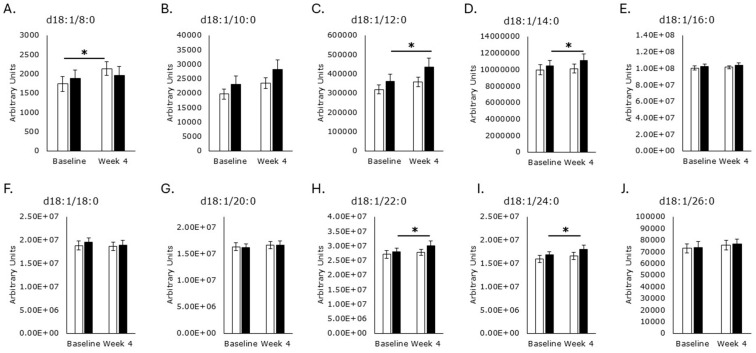
Saturated even acyl chain sphingomyelins. Serum levels of d18:1/8:0 ((**A**); *p* = 0.8), d18:1/10:0 ((**B**); *p* = 0.08), d18:1/16:0 ((**E**); *p* = 0.4), d18:1/18:0 ((**F**); *p* = 0.5), d18:1/20:0 ((**G**); *p* = 0.8), and d18:1/26:0 ((**J**); *p* = 0.2) were unaffected by RPS treatment. Serum levels of d18:1/12:0 ((**C**); *p* = 0.009), d18:1/14:0 ((**D**); *p* = 0.04), d18:1/22:0 ((**H**); *p* = 0.03), and d18:1/24:0 ((**I**); *p* = 0.03) increased in the RPS treatment group. Serum levels of d18:1/8:0 ((**A**); *p* = 0.02) increased in the placebo group, while d18:1/10:0 ((**B**); *p* = 0.09), d18:1/12:0 ((**C**); *p* = 0.09), d18:1/14:0 ((**D**); *p* = 0.4), d18:1/16:0 ((**E**); *p* = 0.3), d18:1/18:0 ((**F**); *p* = 0.7), d18:1/20:0 ((**G**); *p* = 0.5), and d18:1/26:0 ((**J**); *p* = 0.8) were unaffected. White boxes, placebo; Black boxes, RPS; Wilcoxon signed-rank test; Mean +/− SEM; *, *p* < 0.05.

**Figure 6:**
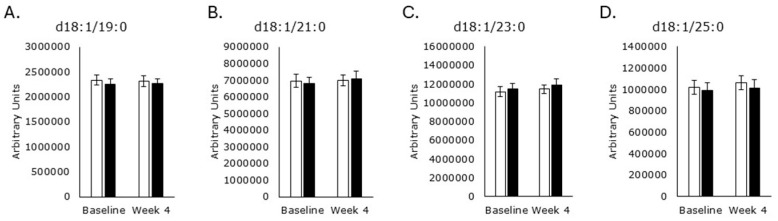
Saturated odd acyl chain sphingomyelins. Serum levels of d18:1/19:0 ((**A**); *p* = 0.8), d18:1/21:0 ((**B**); *p* = 0.1), d18:1/23:0 ((**C**); *p* = 0.3), and d18:1/25 ((**D**); *p* = 0.7) were unaffected by RPS treatment. Similarly, serum levels of d18:1/19:0 ((**A**); *p* = 0.8), d18:1/21:0 ((**B**); *p* = 0.7), d18:1/23:0 ((**C**); *p* = 0.3), and d18:1/25 ((**D**); *p* = 0.4) were unaffected by the placebo. White boxes, placebo; Black boxes, RPS; Wilcoxon signed-rank test; Mean +/− SEM.

**Figure 7:**
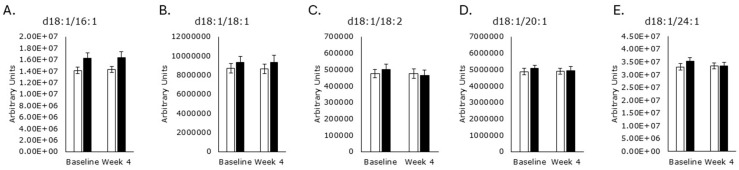
Unsaturated acyl chain sphingomyelins. Serum levels of d18:1/16:1 ((**A**); *p* = 1.0), d18:1/18:1 ((**B**); *p* = 0.9), d18:1/18:2 ((**C**); p = 0.1), d18:1/20:1 ((**D**); *p* = 0.1), and d18:1/24:1 ((**E**); *p* = 0.06) were unaffected by RPS treatment. Serum levels of d18:1/16:1 ((**A**); *p* = 0.6), d18:1/18:1 ((**B**); *p* = 0.4), d18:1/18:2 ((**C**); *p* = 0.7), d18:1/20:1 ((**D**); *p* = 0.8), and d18:1/24:1 ((**E**); *p* = 0.7) were unaffected by the placebo. White boxes, placebo; Black boxes, RPS; Wilcoxon signed-rank test; Mean +/− SEM.

**Figure 8:**
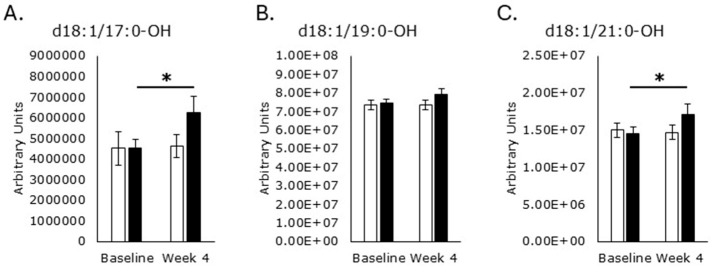
Hydroxylated acyl chain sphingomyelins. Serum levels of d18:1/17:0-OH ((**A**); *p* = 0.01) and d18:1/21:0-OH ((**C**); *p* = 0.04) increased in the RPS group, but the placebo had no effect on d18:1/17:0-OH ((**A**); *p* = 0.7) or d18:1/21:0-OH ((**C**); *p* = 0.8). Neither RPS (*p* = 0.09) nor placebo (*p* = 0.9) affected d18:1/19:0-OH (**B**). White boxes, placebo; Black boxes, RPS; Wilcoxon signed-rank test; Mean +/− SEM; *, *p* < 0.05.

**Figure 9:**
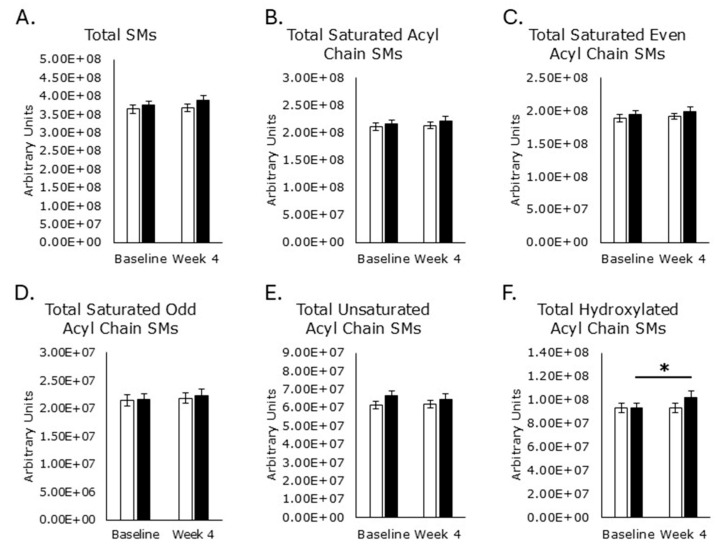
Total sphingomyelin levels by subcategory. Serum levels of total SMs ((**A**); *p* = 0.09), total saturated acyl chain SMs ((**B**); *p* = 0.4), total even acyl chain SMs ((**C**); *p* = 0.4), total odd chain saturated SMs ((**D**); *p* = 0.3), and total unsaturated acyl chain SMs ((**E**); *p* = 0.3) were not affected by RPS treatment. Serum levels of total SMs ((**A**); *p* = 0.4), total saturated acyl chain SMs ((**B**); *p* = 0.2), total even acyl chain SMs ((**C**); *p* = 0.3), total odd chain saturated SMs ((**D**); *p* = 0.5), and total unsaturated acyl chain SMs ((**E**); *p* = 0.9) were not affected by the placebo. Total hydroxylated acyl chain SMs increased in the RPS treatment group (*p* = 0.04) but the placebo had no effect ((**F**); *p* = 0.6). White boxes, placebo; Black boxes, RPS; Wilcoxon signed-rank test; Mean +/− SEM; *, *p* < 0.05.

Choline plays an important role in the synthesis of phospholipids, including phosphatidylcholine and, subsequently, sphingomyelins (Figure 2). While phosphatidylcholine was not targeted for detection for this study, 30 different sphingomyelin forms were detected, consisting of a sphingosine base, phosphocholine head, and acyl tails of varying chain length, saturation, and hydroxylation status (Figure 5, Figure 6, Figure 7, Figure 8 and Figure 9). Levels of saturated even acyl chain SMs d18:1/12:0 (*p* = 0.009; Figure 5C), d18:1/14:0 (*p* = 0.04; Figure 5D), d18:1/22:0 (*p* = 0.03; Figure 5H), and d18:1/24:0 (*p* = 0.03; Figure 5I) were increased in the RPS group while the placebo increased levels of d18:1/8:0 (*p* = 0.02; Figure 5A).

Saturated odd acyl chain SMs were unaffected by either treatment (Figure 6), as were SMs with unsaturated acyl chains (Figure 7).

Hydroxylated acyl chain SMs d18:1/17:0-OH (*p* = 0.01; Figure 8A) and d18:1/24:0-OH (Figure 8C) were increased in the RPS group while the placebo had no effect.

Total SMs (Figure 9A), total saturated acyl chain SMs (Figure 9B), total saturated even-carbon acyl chain SMs (Figure 9C), total saturated odd acyl chain SMs (Figure 9D), and total unsaturated acyl chain SMs (Figure 9E) were unaffected by treatment, but total hydroxylated acyl chain SMs (Figure 9F) were elevated in the RPS treatment group. Placebo treatment had no effect on any of these SM groups (Figure 9).

### 3.5. Sphingomyelinase and Phospholipase Activity

**Figure 10:**
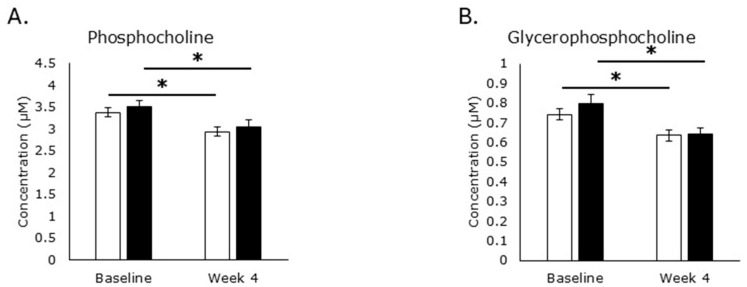
Sphingomyelin catabolism metabolites. Serum levels of phosphocholine decreased in the RPS (*p* = 0.007) and placebo treatment groups (*p* = 0.0001; (**A**)) and glycerophosphocholine levels (**B**) decreased in both the RPS (*p* = 0.0003) and placebo (*p* = 0.0003) treatment groups. White boxes, placebo; Black boxes, RPS; Wilcoxon signed-rank test; Mean +/− SEM; *, *p* < 0.05.

Increases in sphingomyelinase activity, producing phosphocholine from SM, or lysophospholipase activity, producing glycerophosphocholine from phosphatidylcholine, might contribute to elevated serum choline levels because both metabolites can be further degraded to choline (Figure 2). However, levels of phosphocholine were decreased in both the RPS (*p* = 0.007) and placebo (*p* = 0.0001) treatment groups (Figure 10A). Levels of glycerophosphocholine were also decreased in both the RPS (*p* = 0.0003) and placebo (*p* = 0.0003) treatment groups (Figure 10B), suggesting that catabolic activity on endogenous phospholipids cannot explain the elevated serum choline levels in RPS consuming individuals.

## 4. Discussion

Serum choline levels were elevated in the RPS treatment group after four weeks and this effect was positively correlated with changes in intestinal barrier-associated *Akkermansia*. Both RPS and placebo treatments led to reductions in TMA, but neither affected TMAO levels. RPS did not affect betaine levels or down-stream metabolites, suggesting that the choline increases were not due to changes in choline oxidation or one-carbon metabolism. Similarly, RPS did not influence acetylcholine or acetate levels, indicating that increased choline levels are not due to decreased acetylcholine formation or increased acetylcholinesterase activity. RPS consumption led to increases in certain forms of sphingomyelins, which are phospholipids containing choline, including those with saturated even acyl chains and hydroxylated acyl chains. Taken together, these findings suggest that consumption of RPS does not increase TMAO levels, but rather enhances choline absorption and promotes the incorporation of choline into sphingomyelins.

Resistant starches from different sources have been reported to have different effects on TMAO production. Supplementing the diet with high amylose maize starch led to increases in TMAO in a healthy population [33], but banana RS2 did not influence TMAO or choline levels in hemodialysis patients [34]. Despite an increase in serum choline, RPS consumption led to decreased serum levels of TMA and had no effect on serum TMAO levels. Others have reported that strict paleolithic diets, which contain very low levels of RS types 2 and 3, promote higher levels of TMAO [41], though this effect was not significant in women [42]. The discrepancies between studies may reflect previously unappreciated differences between high amylose maize starch, banana RS2, and RPS. Given that RS and RPS seem to play protective roles in the context of choline metabolism, coadministration of choline with these forms of resistant starch might mitigate the adverse effects associated with choline supplements like alpha-glycerophosphocholine [43]. It was also noted that both treatments decreased the levels of TMA, the microbial precursor of TMAO. Introduction of prebiotic carbohydrates is known to reduce protein fermentation metabolites [36], and choline is typically abundant in protein-containing foods, so it is possible that both RPS and placebo shifted the fermentation profile of the microbiota, despite the placebo having to over effects on the composition of the gut microbiota [36].

Serum choline comes from three main sources: digestion and absorption of choline from foods, endogenous synthesis in the liver, and cleavage from choline-containing phospholipids present in cell membranes [44]. However, de novo synthesis of choline in the liver is inadequate to meet human physiological requirements, making choline an essential nutrient [1]. Positive correlations between choline and increases in *Akkermansia* levels in the gut of the RPS consuming group suggest that increased serum choline levels are due to enhanced intestinal absorption, which reduces the availability of choline to gut bacteria, potentially explaining the reduction in TMA levels. This hypothesis is consistent with RPS consumption reducing serum histamine levels that suggested intestinal barrier function [30], and increased serum levels of diet-derived, lipid soluble vitamins retinol and α-tocopherols reflecting improved intestinal absorption [45]. However, it is unlikely that increased SM absorption from the diet can explain increased SM levels in the diet because supplementation with SM does not lead to increased serum SM levels [25].

Oxidation of choline into betaine and further metabolism of betaine into dimethylglycine and sarcosine was unaffected. Similarly, RPS did not alter acetylcholine synthesis or metabolism. Consumption of RPS did lead to increased levels of SM, a heterogeneous group of phospholipids assembled through a complex series of steps. Choline is phosphorylated by choline kinase in the first committed step towards synthesizing phosphatidyl choline [20], but phosphocholine is also generated during the degradation of sphingomyelin by various sphingomyelinases [46]. While phosphatidyl choline levels were not captured in our analysis, phosphocholine levels were reduced in both treatment groups, suggesting that increased choline levels are not due to increased sphingomyelinase activity. Similarly, glycerophosphocholine is a degradation product of phosphatidylcholine and levels of this metabolite were reduced in both treatment groups, suggesting the RPS is not increasing choline levels by enhancing lysophospholipase activity and phosphatidylcholine degradation [47]. Collectively, these data support the conclusion that increased levels of SM are a consequence of the functional incorporation of choline whose levels increased due to enhanced absorption from the diet in RPS consuming individuals.

RPS consumption increased the serum concentrations of a subset of SMs, specifically those with saturated and hydroxylated acyl chains. We previously reported that free fatty acid (FFA) levels were significantly decreased in this same population, an effect that did not appear to differ among the different FFAs detected [32]. Reductions in FFA levels were robust and attributed to reduced FFA release from adipocytes [32], but it is possible that reductions in some FFA might reflect their incorporation into SMs. While the hydroxylated FFAs corresponding to the hydroxylated acyl chains of SM that were increased in RPS consuming individuals (FA17:0-OH, FA19:0-OH, and FA21:0-OH) were not detected, levels of the saturated acyl chains present in the SM forms that increased (FA10:0, FA12:0, FA14:0, FA22:0, and FA24:0) were among the unchanged FFAs in the RPS treatment group [32]. This suggests that RPS-dependent increases in SMs did not influence the abundance of FFAs and that FFA reductions are likely a due to enhanced retention by adipocytes.

Myelin sheaths contain relatively high amounts of hydroxylated SMs and long-chain SMs [48], and while mice lacking the enzyme required for SM hydroxylation develop normally, they experience enhanced myelin sheath degeneration and central nervous system dysfunction [24,49]. Dietary sources of choline are important for pregnant women and nursing mothers in part because this nutrient supports normal brain development, including myelination [6]. A study in Brazilian women found that serum levels of hydroxylated SMs during pregnancy were associated with lower anxiety scores during pregnancy and their first year post-partum [50]. Similarly, high levels of baseline hydroxylated SMs and increases in hydroxylated SMs were correlated with depression recovery in treatment-seeking depressed participants [51]. These studies suggest that maintaining higher SM levels is important throughout life, not just during brain development and maturation. Supplementing the diet with RPS may support choline incorporation into SM species particularly important for neuronal development, such as hydroxylated SMs.

## 5. Conclusions

RPS supplementation increased dietary choline absorption, an effect that was positively correlated with increases in *Akkermansia* in the gut microbiome. This observation is consistent with other RPS investigations supporting improved intestinal barrier function and nutrient absorption. Levels of saturated even acyl chain and hydroxylated acyl chain SMs were elevated while makers of SM and phosphatidyl choline degradation were decreased, suggesting that increased SM levels follow from enhanced absorption of choline. Levels of the choline metabolite TMA were reduced and levels of TMAO, the cardiovascular toxin produced from TMA, were unaffected by RPS. Therefore, RPS supplementation may offer a preferable way to enhance choline levels while mitigating the microbiome-associated risks associated with this nutrient. Studies examining choline co-administered with RPS are warranted.

## 6. Patents

MSP Starch Products Inc. sister company McPharma Biotech Inc. holds relevant patents US11058711B2, CA3024201A1, AU2017294806A1, and provisional patent applications 63/358,194 and 63/728,887.

## Data Availability

The data presented in this study are available on request from the corresponding author, but the data are owned by MSP Starch Products Inc. and restrictions apply to the use of these data, including the execution of nondisclosure agreements and/or material transfer agreements.

## Abbreviations

The following abbreviations are used in this manuscript:

BMI	Body mass index
CVD	Cardiovascular disease
FFA	Free fatty acid
LC-MS/MS	Liquid chromatography coupled to tandem mass spectrometry
NAFLD	Non-alcoholic fatty liver disease
QC	Quality control
RPS	Resistant potato starch
RS	Resistant starch
RS2	Resistant starch type 2
SM	Sphingomyelin
SPT	Serine palmitoyltransferase
TMA	Trimethylamine
TMAO	Trimethylamine oxide
